# Compound K Production: Achievements and Perspectives

**DOI:** 10.3390/life13071565

**Published:** 2023-07-14

**Authors:** Luan Luong Chu, Nguyen Trinh Yen Hanh, My Linh Quyen, Quang Huy Nguyen, Tran Thi Phuong Lien, Khanh Van Do

**Affiliations:** 1 Faculty of Biotechnology, Chemistry and Environmental Engineering, Phenikaa University, Hanoi 12116, Vietnam; 2 Faculty of Biology, University of Science, Vietnam National University, Hanoi (VNU), 334 Nguyen Trai, Thanh Xuan, Hanoi 10000, Vietnamnguyenquanghuy@vnu.edu.vn (Q.H.N.); 3 National Key Laboratory of Enzyme and Protein Technology, University of Science, Vietnam National University, Hanoi (VNU), 334 Nguyen Trai, Thanh Xuan, Hanoi 10000, Vietnam; 4 Faculty of Biology and Agricultural Engineering, Hanoi Pagadogical University 2, Vinh Yen City 283460, Vietnam; tranthiphuonglien@hpu2.edu.vn; 5 Faculty of Biomedical Sciences, Phenikaa University, Hanoi 12116, Vietnam

**Keywords:** Compound K (CK), *Panax ginseng*, β-glucosidase, endophytes, *Saccharomyces cerevisiae*

## Abstract

Compound K (CK) is one of the major metabolites found in mammalian blood and organs following oral administration of *Panax* plants. CK, also known as minor ginsenoside, can be absorbed in the systemic circulation. It has garnered significant attention in healthcare and medical products due to its pharmacological activities, such as antioxidation, anticancer, antiproliferation, antidiabetics, neuroprotection, and anti-atherogenic activities. However, CK is not found in natural ginseng plants but in traditional chemical synthesis, which uses toxic solvents and leads to environmental pollution during the harvest process. Moreover, enzymatic reactions are impractical for industrial CK production due to low yield and high costs. Although CK could be generated from major ginsenosides, most ginsenosides, including protopanaxatriol-oleanane and ocotillol-type, are not converted into CK by catalyzing β-glucosidase. Therefore, microbial cell systems have been used as a promising solution, providing a safe and efficient approach to CK production. This review provides a summary of various approaches for the production of CK, including chemical and enzymatic reactions, biotransformation by the human intestinal bacteria and endophytes as well as engineered microbes. Moreover, the approaches for CK production have been discussed to improve the productivity of target compounds.

## 1. Introduction

The genus *Panax*, belonging to the family *Araliaceae*, is known as one of the most popular herbal medicines in East Asia and North America. *Panax* plants, including *Panax ginseng* C.A. Meyer (Korean ginseng), *Panax japonicus* C.A (Japanese ginseng), *Panax quinquefolius* L. (America ginseng), *Panax notoginseng* (Burkill) F.H. Chen (Sanchi ginseng), have been widely used as dietary supplements in recent years [1,2]. There are various chemical components in ginseng, such as ginsenosides, polysaccharides, polyacetylenes, glycoconjugate compounds, and amino acids. Among these, ginsenosides play a role as a major constituent and are responsible for the diverse biological and pharmacological activities of ginseng [3,4]. To date, more than 150 different types of ginsenoside have been isolated and identified from the roots, fruits, flower buds, and leaves of ginseng, and other medicinal plants. Ginsenosides, also known as triterpene saponins, are represented by Rx. While the “R” is expressed for the root, the “X” is determined by the chromatographic polarity in alphabetical order: the most polar is determined by A, and the least polar is marked by H. In general, a chemical component of most ginsenosides contains a dammarane skeleton (17 carbons in a four-ring structure) and a sugar moiety attached to the C-20 and C-3 positions (arabinose, glucose, rhamnose, and xylose) [5,6]. According to their backbone skeletons, ginsenosides can be categorized into four groups: protopanaxadiol (PPD), protopanaxatriol (PPT), oleanolic acid (OA), and ocotillol-type. PPD has only a dammarane backbone (for example, Ra1-3, Rb1-3, Rc, Rd, Rg3, Rh2, and Rs1), while PPT has an additional hydroxyl group at the C-6 position (for example, Re, Rf, Rg1-2, and Rh1). Moreover, the pentacyclic triterpenoid base is a characterization of OA type (Ro) whereas ocotillol-type ginsenosides possess a five-membered epoxy ring at the C-20 position (Pseudoginesnoside F11 and Majonoside R1-2) (Table 1) [7,8]. Along with the isolation and identification of ginsenosides from plants, extensive research has been conducted to investigate and understand the biological mechanisms of ginsenosides. Ginsenosides exhibit various beneficial effects on human health, including antimicrobial, antioxidant, anticancer, antitumor, and anti-proliferative activities [9,10]. Furthermore, ginsenosides have been demonstrated to enhance the central nervous system and protect the blood vessels from cardiovascular disease [11,12]. Recently, ginsenosides and their derivatives have been used as cosmetic and dietary supplements [2,13]. Interestingly, ginsenosides have shown potential as promising medicines for the treatment and prevention of SARS-CoV-2 [14].

**Figure 1:**
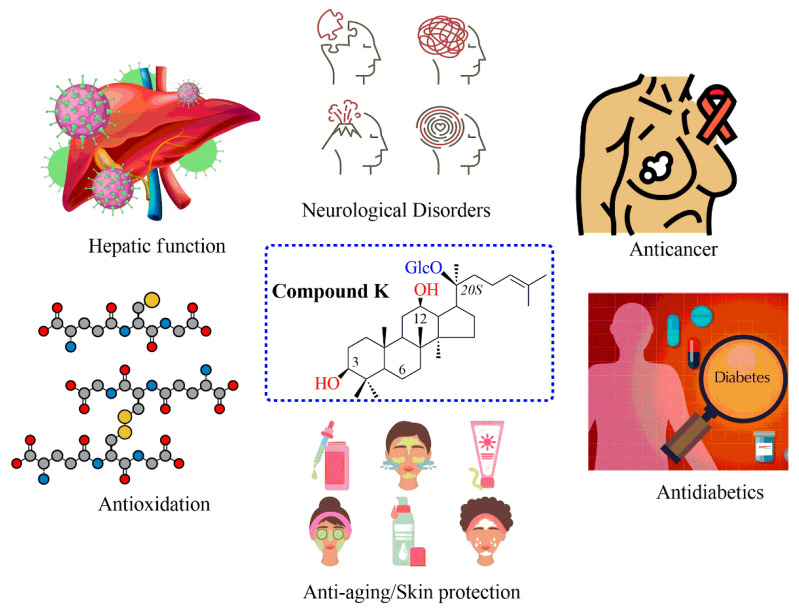
The chemical structure of CK and its biological activities.

Compound K is one PPD type of ginsenoside for application to human health. CK has a molecular weight of 622.86 g mol^−1^ and the chemical formula C_36_H_62_O_8_. The IUPAC name of CK is (2S,3R,4S,5S,6R)-2-[(2S)-2-[(3S,5R,8R,9R,10R,12R,13R,14R,17S)-3,12-dihydroxy-4,4,8,10,14-pentame-thyl-2,3,5,6,7,9,11,12,13,15,16,17-dodecahydro-1H-cyclopenta[a]phenanthren-17-yl]-6-methylhept-5-en-2-yl]oxy-6-(hydroxymethyl)oxane-3,4,5-triol. CK is also known as 20-O-β-D-glucopyranosyl-20(*S*)-protopanaxadiol, compound M1-O, ginsenoside M1, and GM1 saponin [15]. CK is rarely found in natural ginseng but through the transformation of major ginsenosides. Bioconversion of CK products has been reported through deglycosylated reactions by human intestinal bacteria and endophytes [16]. Enzymatically synthesized and metabolically engineered yeasts have emerged as alternative approaches to producing CK in recent years [17,18]. Importantly, CK exhibits significant biological and pharmaceutical properties in different aspects, such as antioxidation, antiproliferation, protecting organs, cognitive, central nervous systems, and anticancer activities, which have been reported over the decades. In addition, CK also plays a significant role in hepatic function and antidiabetic disease (Figure 1) [19,20]. Furthermore, while a small quantity of CK is adequate for therapeutic applications, it is insufficient for the development of novel drugs for the management of a variety of diseases. As a result, in order to create medicine, a sizable amount of CK must be used. In this review, we summarize the research progress on the biological effects of CK and provide an update on the biosynthesis of CK from microbial biotransformation, enzymatic synthesis, and metabolically engineered microbes over the past decade. The advantages and disadvantages of each approach have been discussed for future perspectives in CK production.

## 2. Biological Significance in Humans

**Figure 2:**
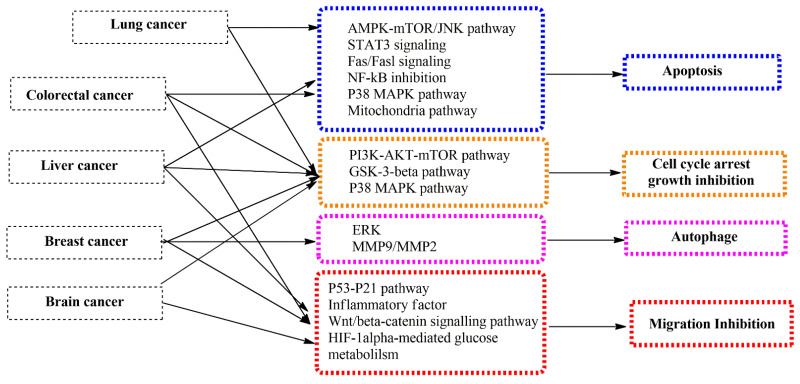
The functional mechanism of CK on different cancer cell lines. Abbriviation: AMPK, adenosine monophosphate protein kinase; Akt, protein kinase B; GSK3β, glycogen synthase kinase; HIF, hypoxia inducible factor; JNK, c-Jun N-terminal kinase; MAPK, mitogen-activated protein kinase; MAPK, mitogen-activated protein kinase; MMP, metalloproteinase; mTOR, mammalian target of rapamycin; PI3K, phosphatidylinositol 3-kinase; ROS, reactive oxygen species; STAT, signal transducer and activator of transcription.

CK is known as a minor ginsenoside. Compared to other ginsenosides, CK has higher and more diverse pharmacological applications in some respects. There are numerous studies on the pharmaceutical properties of CK in different aspects, such as protecting organs, cognitive and central nervous systems, antioxidation, anticancer, antiproliferation, and antidiabetics (Figure 1) [21,22]. Among these pharmaceutical activities, anticancer agents have been the most important aspect of CK. According to the Global Cancer Observatory (GLOBOCAN), there were an estimated 19.3 million cancer cases worldwide in the year 2020. It is projected that the global burden of cancer cases will be 29.4 million in the year 2040, based on the aging and growing population. Therefore, recently, there have been more studies about the discovery of new drugs for cancer treatments. The demonstration of the cell’s biological mechanism is necessary to develop novel drugs for the treatment of cancer cell lines. According to recent research, CK exhibits anticancer activity via multiple molecular mechanisms, such as apoptosis, inhibitory apoptotic proteins, regulation of cell growth, impact on cell invasion and metastatic activity, and autophagy. It is reported that the effect of CK on cancer cells is related to the AMPK-mTOR/JNK pathway, the PI3K/Akt/mTOR pathway, and reactive oxygen species (ROS) (Figure 2) [19]. For example, CK has been shown to inhibit NF-κB by hindering Annexin A2, a protein associated with cancer, from binding to the NF-κB p50 subunit. NF-κB acts as a crucial regulator in the development of human astroglial cells and liver cancer cells, primarily by inhibiting apoptosis proteins [23]. Furthermore, CRISPR/cas technology has been applied to inhibit p-STAT3 in human liver cancer cells. As a result, apoptosis of liver cancer in HepG2 cells is activated when CK is present. In another example, the anticancer ability of CK was examined in the HER2-positive breast cancer cell line (SKBR3) and HER2-negative breast cancer cell line (MDA-MB-231) via 3-(4-5-dimethylthiazol-2-yl)-2-5-diphenyltetrazolium bromide assays, propidium iodide, annexin V staining, and morphological changes. The results indicated that CK, by controlling protein kinase B or Akt activity, could exert anticancer effects and be employed as a medicinal component for breast cancer [24]. It is reported that CK induces apoptosis in lung cancer cell lines via the AMPK-mTOR and JNK pathways. In the case of colon cancer, CK induced autophagy and apoptosis through the generation of reactive oxygen species and the activation of JNK. In another study of glioblastoma cells, CK significantly inhibited the growth and metastasis of these cells via the PI3K/Akt/mTOR pathway. It can be seen that CK inhibits different cancer cell lines through various pathways (Figure 2) [19].

Several studies have shown the positive impacts of CK on hepatic function, such as suppressing liver damage by preventing c-Jun N-terminal kinase signaling in HepG2 cells and also protecting the liver from sodium valproate-induced hepatotoxicity [25]. Furthermore, CK has shown a significant decrease in nitric oxide levels, which play an important role in the pathophysiological alterations of inflammatory disorders, at concentrations higher than 10 μg mL^−1^. CK has also been proven to have a better ability to prevent inflammation compared to other ginsenosides. CK reduces inflammation in lipopolysaccharide-treated RAW 264.7 cells by lowering the production of proinflammatory cytokines such as tumor necrosis factor-α, Interleukine (IL)-1β, and IL-6 [26]. In other research, a CK-rich fraction was developed and demonstrated to suppress nitric oxide production on lipopolysaccharide-treated RAW 264.7 cells, lower mRNA levels of inducible nitric oxide synthase and interferon-β, and inhibit nuclear factor-kappa B (NF-κB) transcriptional progress [27].

In anti-diabetic research, CK has also shown remarkable activity. It has been demonstrated to improve insulin secretion triggered by glucose. In a different study, a rat model of type 2 diabetes mellitus with insulin resistance was used to test the anti-diabetic effects of CK, and the results showed that CK could increase food intake, body weight, insulin sensitivity, and fasting serum insulin level in diabetic rats [28]. Another notable pharmaceutical application of CK is neuroprotection. CK has shown considerable pharmacological effects on the central nervous system. Studies have explored the application of CK in the treatment of neurological disorders such as depression, Alzheimer’s disease, Parkinson’s disease, and more [29]. In the prefrontal cortex and hippocampus of chronic unpredictable mild stress rats, CK boosted levels of 5-hydroxytryptamine, dopamine, and their metabolites, enhanced the activity of glutathione and glutathione peroxidase, and also counteracted MAO-B overexpression in these regions. Therefore, CK treatment led to increased brain-derived neurotrophic factor and nerve growth factor expression, demonstrating its antidepressant properties in rodents [30]. CK has also shown positive effects on vascular dementia by reducing the Amyloid β1-42 deposition caused by chronic cerebral hypoperfusion and improving cognitive impairment through the upregulation of pSer9-Glycogen synthase kinase 3β (pSer9-GSK3β) and the insulin degrading enzyme (IDE) [31].

CK has been linked to anti-atherogenic activities. CK and its derivatives have shown the ability to activate the liver X receptor alpha (LXRα) and attenuate the development of atherosclerosis in ApoE-/-mice [32]. CK also regulates the reverse transport of cholesterol and promotes the ATP-binding cassette transporter A1 (ABCA1), resulting in a reduction in total cholesterol in the blood, blood viscosity, and relieving atherosclerosis [15]. In terms of anti-aging, CK has been used as a cosmetic ingredient. It enhances the production of hyaluronic acid by activating Src (tyrosine kinase)-dependent Akt and extracellular signal-regulated kinase (ERK). Furthermore, CK reduces the production of cyclooxygenase-2 and matrix metalloproteinase-1 in ultraviolet B irradiated NIH-3T3 skin fibroblast cells or tumor necrosis factor-alpha-stimulated cells and restores the expression level of type I collagen [33]. This research indicates that CK plays an important role in anti-aging activities. Interestingly, it is predicted that CK or/and another ginsenoside might inhibit myocardial injury by SARS-CoV-2 [34].

## 3. Biosynthesis Approaches for Compound K

### 3.1. Ginseng Extraction and Chemical Synthesis

Extraction from ginseng and chemical synthesis are two traditional approaches for the synthesis of various ginsenosides [35,36,37]. While the major ginsenosides, such as Rb1, Rb2, Rb3, Rc, and Rd, are present in different parts of ginseng plants at different ages, CK is not naturally present in ginseng [38]. Although the cultivated areas of ginseng are expanding around the world, low productivity is still the major challenge in extraction for CK production [39]. Production of minor ginsenosides has been obtained through mild acid hydrolysis and alkaline cleavage, but the chemical synthesis of CK is rare [40,41]. The disadvantage of chemical synthesis approaches is the requirement for multiple components with low yields. Moreover, these chemical synthesis methods generate non-selectively hydrolyze sugar moieties and produce by-products, resulting in a decrease in the amount of ginsenosides. Furthermore, one of the major shortcomings of these chemical processes is their contribution to environmental pollution through the emission of carbon dioxide [42,43].

### 3.2. Enzymatically Synthesized CK

Enzymatic synthetic methods are conventional approaches to produce CK. Enzymatic synthesis displays higher region-specific activity in comparison with chemical synthesis. In these methods, the major ginsenosides, including Rb1, Rb2, Rb3, and Rd, are converted to CK using crude enzymes or purified enzymes [44].

#### 3.2.1. Enzyme from Native Microbes

**Figure 3:**
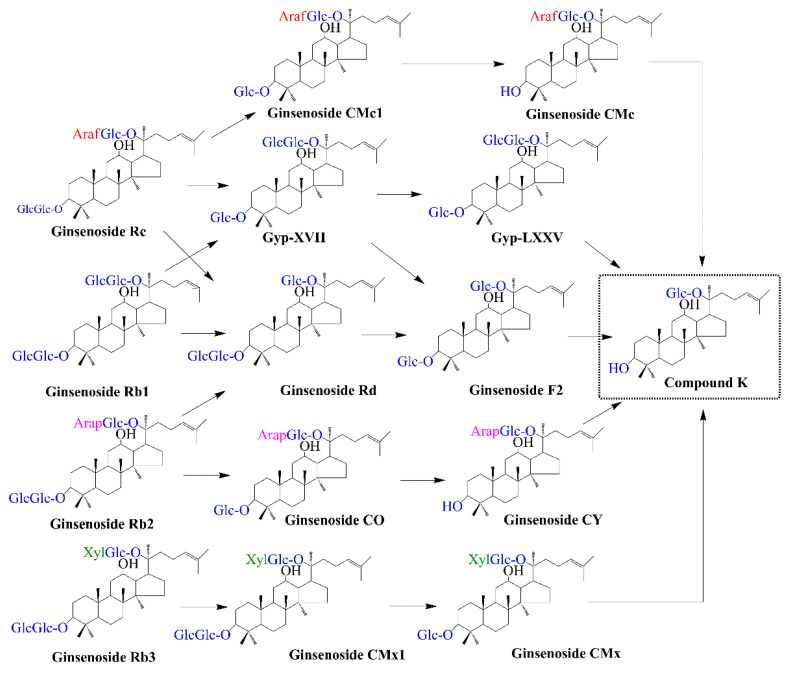
Biotransformation pathway for CK production by enzymatic reaction and microbes.

Various types of glucosidases (EC 3.2.1), consisting of β-D-glucosidase (3.2.1.2), β-D-xylosidase (EC 3.2.1.37), α-L-rhamnosidase (EC 3.2.1.40), β-galactosidase (EC 3.2.1.23; lactase), and glycoside hydrolases, are responsible for the hydrolytic reaction [45]. Due to its easy preparation under mild conditions, crude enzyme is the general trend for ginsenoside conversion. For example, crude enzymes can be prepared from *Lactobacillus* sp. [46,47,48], *Penicillium* sp. [49], medicinal mushroom *Stereum hirsutum* [50], *Aspergillus* sp. [51,52], or *Fomitella fraxinea* [53]. Three β-glucosidase active bacterial strains isolated from traditional Korean fermented food (Kim Chi) were used for the conversion of Rb1 or Rd to CK. The optimal time for an enzymatic reaction was 72 h at a pH of 6.0 to 8.0 and a temperature of 30 °C. The conversion percentages from Rb1 to CK were around 99%, and 97% of Rd was decomposed to CK under optimal conditions [46,47,48]. Notably, the hydrolysis of the PPD-type saponin mixture showed the ability to form CK by using crude glycoside hydrolase. A crude preparation of β-galactosidase from *Aspergillus oryzae* was found to produce CK, whereas crude lactase from *Penicillium* sp. showed activity for conversion to CK from a PPD-type saponin mixture [49]. Similarly, *A. niger* XD101, which produces the ginsenoside-hydrolyzing β-glucosidase, transformed Rb1 via the following pathways: Rb1 → Rd → F2 → CK at pH 4–5 and a temperature of 50–60 °C in 72 h, and resulting in a high conversion yield of 94.4% [51]. β-glucosidase-producing *Stereum hirsutum* JE0512, sourced from wild ginseng, was used to produce CK from ginseng extracts in solid-state fermentation using 20 g of corn bran as a substrate. Various biotransformation approaches were identified to produce CK from major ginsenosides, such as Rb1 → Rd → F2 → CK, Rc → Gyp XVII → Gyp LXXV → CK, Rb2 → CO → CY → CK, and Rb3 → CMx1 → CMx → CK (Figure 3; Table 2) [50]. Although the conversion yield of CK is high, crude enzymes require a large quantity of saponin mixture and exhibit low productivity.

Recently, enzymatic catalysis has focused on using purified enzymes to produce CK from major ginsenosides [54]. Purified and characterized enzymes not only provide extremely selective reactions under very mild conditions but also efficiently remove by-products. Purified enzymes can be obtained from hydrolase-producing microorganisms or recombinant microbes. In the former approach, filamentous fungi are known as one of the most widely produced species of cellulolytic enzymes [55]. For example, ginsenoside type I and β-glucosidase were isolated and purified from *Aspergillus* sp. g48p and *Paecilomyces Bainier*. The β-glucosidase from *Paecilomyces Bainier* shows only the ability to convert Rb1 to CK at pH 3.5 and 60 °C. By contrast, ginsenoside type I from *Aspergillus* sp. g48p can hydrolyze multi-glycosides of PPD to produce F2, CK, and Rh2. It can hydrolyze Rb1, Rb2, Rb3, Rc, and Rd at β-glucoside of C-3; hydrolyze Rb1 at β-glucoside of C-20; hydrolyze Rb2 at α-arabinoside of C-20; hydrolyze Rb3 at β-xyloside; and hydrolyze Rc at α-arabinoside (Figure 3; Table 2) [56,57]. In another case, enzyme preparation from cultured mycelia of *Armillaria mellea* can convert Rb1 and Rb2 to CK with high yields and without food safety issues [17,33]. Interestingly, there are several commercial glycoside hydrolases available on the market. Almost all of these enzymes are isolated from fungi, which are used to hydrolyze Korean red ginseng. For example, β-glucanase in Ultraflo L is isolated from *Humicolar insolens*. Moreover, cellulase, xylanase, hemicellulose, and β-glucanase were mixed in Viscozyme, which is sourced from *Aspergillus* sp. In another case, pectinase, hemicellulose, and cellulase isolated from *A. niger* and *Trichoderma longibrachiatum* are packaged in rapidase [49,58]. Naringinase (flavonoid β-D-glucosidase) can be used to hydrolyze ginsenoside to generate CK from G-IV, G-IV → G-VIII → G-XII → CK [59]. The use of commercial enzymes has resulted in an increased rate of bioconversion and extraction.

#### 3.2.2. Recombinant Enzymes

Because recombinant enzymes are expressed under controlled conditions, host microbes can produce the highest concentration of enzymes. Moreover, recombinant microorganisms easily address several environmental effects. As a result, microbes harboring recombinant enzymes not only exhibit the significant efficiency of biotransformation to CK but also reduce costs and processing time. *Escherichia coli* (*E. coli*) is known as a ubiquitous microbial host for heterologous gene expression due to its favorable growth conditions and well-characterized genetics and physiology [60]. Therefore, alternative sources of recombinant β-glucosidase have been considerably overexpressed in *E. coli* for the production of CK. The recombinant β-glucosidase can be isolated from bacteria, fungi, and archaea. For example, a recombinant β-glucosidase cloned from *Fusobacterium* K-60 showed conversion of ginsenoside Rb1 into CK [61]. In 2010, a novel β-glucosidase from a new strain of *Terrabacter ginsenosidimutants* (Gsoil 3082^T^) was also applied to produce CK. This enzyme showed its function in the transformation pathway as Rb1 → GypXVII → GypLXXV → CK [62]. Interestingly, β-glucosidase derived from *Microbacterium esteraromaticum* was found in two different transformation pathways for the production of CK from ginsenosides: Rb1 → Rd → CK and Rb2 → CY → CK (Figure 3; Table 2) [63,64]. In another case, a novel β-glucosidase from *Bifidobacterium breve* ATCC 15700 was able to produce CK from Rd via F2 [65]. Theoretically, these enzymes exhibit high selectivity and efficiency in hydrolyzing the outer glucose moiety attached to the C-20 position and/or the inner glucose moiety attached to the C-3 position of ginsenosides. While ginsenoside Rb1 and Rb2 were converted to CK by hydrolyzing glucose at both C-3 and C-20 positions, ginsenoside Rd was converted to F2 through the hydrolysis of glucose at the C-3 position. Then, hydrolysis of the C-3 glycoside of F2 produced CK [63,64]. Notably, *E. coli* is also used as a microbial platform for the overexpression of β-glucosidase from *Archaea.* β-glucosidases show a board specificity as they can catalyze the hydrolysis of glycosidic bonds between two and digest glycoside linkages between a sugar and the aglycone.

A thermostable recombinant β-glucosidase from *Sulfolobus solfataricus* exhibited ginsenoside-hydrolyzing activity. It was demonstrated that this enzyme has concomitant β-glucosidase, β-galactosidase, and β-xylosidase activities. Its catalysis converts ginseng root extract to CK through two transformation pathways, namely Rb1 or Rb2 → Rd → F2 → CK and Rc → Mc → CK [66]. In another case, thermostable β-glucosidase from *Sulfolobus acidocaldarius* was found to have the ability to convert major ginsenosides to CK via two pathways (Rb1 → Rd → CK and Rb2 → CY → CK), while β-glucosidase from *Pyrococcus furiosus* showed the hydrolyzing of Rb1, Rb2, and Rc to CK via Rd [66,67]. These enzymes, being found in hyperthermophilic bacteria, exhibited optimal hydrolyzing activity at pHs from 5.0 to 6.0 and temperatures over 80 °C. Interestingly, the combination of two or three sugar hydrolyzing enzymes showed the ability to completely convert ginsenosides Rc, Rb2, and major protopanaxadiol ginsenosides to CK. In particular, the α-L-arabinofuranosidase and β-galactosidase isolated from *Caldicellulosiruptor saccharolyticus* could hydrolyze α-L-arabinofuranoside and β-D-arabiopyranoside, respectively. Moreover, β-glucosidase isolated from *Sulfolobus acidocaldarius* showed the ability to hydrolyze β-D-glucopyranoside. The combination of α-L-arabinofuranosidase and/or β-galactosidase with β-glucosidase results in the production of CK with productivities of 388, 328, and 144 mg L^−1^ h^−1^ from Rc, Rb2, and major protopanaxadiol ginsenosides in ginseng root extract, respectively [68]. Similarly, the combination of α-L-arabinofuranosidase and/or β-galactosidase with β-glucosidase from *Aspergillus tubingensis* KCTC 14166 produced 2.47 g L^−1^ of CK from American ginseng extract [69]. These findings suggest that the combination of several enzymes provides a promising approach to improving the productivity of CK.

**Table 2:** Summary of CK production enzymatic reaction and microbes.

Strains	Transformation Pathway/Products	Buffer/ Medium	Temperature (Degree, °C)	pH	Time (Hours, h)	Titer/Efficiency	Ref.
β-glucosidase from native microbes							
*Armillaria mellea*	Rb1 → XVII/ Rd → F2 → CK	SA	45–55	4.0–4.5	72–96	0.42 (mg mL^−1^)	[17]
*Armillaria mellea* KACC 50013	Rb2 → Rd → F2 → C-K, Rb2 → CO → CY → CK	SA	45	4.5	96	N/A	[38]
*Talaromyces purpureogenus*	Rb1 → Rd → F2 → CK	MDES	60	4.5	48	80.60%	[44]
*Lactobacillus pentosus* DC10	Rd → F2 → CK	SD	30	7	72	97%	[46]
*Leuconostoc mesenteroides* DC102	Rb1 → XVII/Rd → F2 → CK	SD	30	6.0–8.0	72	99%	[47]
*Leuconostoc citreum* LH1	Rb1 → Rd → F2 → CK	SD	30	6	72	99%	[48]
*Stereum hirsutum* JE0512	Rb 1 → Rd → F2 → CK, Rc → Gyp XVII → Gyp LXXV → CK, Rb 2 → CO→ CY → CK, Rb 3 → CMx1 → CMx → CK	PDA	25	6.8	10 days	54.48 (mg g^−1^)	[50]
*Aspergillus niger* XD101	Rb1 → Rd → F2 → CK	AB	50-60	4–5	72	94.4%	[51]
*Aspergillus tubingensis* KCTC 14166	Rc → Mc1 → Mc → CK Rb1 → Rd → F2 → CK Rb2 → CO → CY → CK	CB/PB	55	4.0	20	418 (mg L^−1^ h^−1^)	[52]
*Fomitella fraxinea*	Rb1 → Rd → F2 → CK Rc → Rd → F2 → CK Rc → CMc1 → CMc → CK	AB	45	4.5	8	N/A	[53]
*Paecilomyces Bainier* sp. 229	Rb1 → Rd → F2 → CK	FB	45	3.5	24	84.30%	[56]
*Aspergillus niger* g.848	Rb1 → Rd → F2 → CK	AB	45	5	18	69.5%	[57]
Naringinase							
*Gynostemma pentaphyllum*	G-IV→ G-VIII → G-XII → CK (theory)	AB	50	4.1	71	65.44%	[59]
α-L-arabinofuranosidase and/or β-galactosidase with β-glucosidase							
α-L-arabinofuranosidase and β-galactosidase from *Caldicellulosiruptor saccharolyticus*; β-glucosidase from *Sulfolobus acidocaldarius*	Rc → Mc/Rd → CK Rb1 → Rd → CK Rb2 → Rd/CY → CK	CB/PB	75	6.0	12	388 (mg L^−1^ h^−1^)	[68]
					14	328 (mg L^−1^ h^−1^)	
					20	144 (mg L^−1^ h^−1^)	
*Aspergillus tubingensis* KCTC 14166	Rb1 → Rd → F2 → CK, Rb2 → CO/Rd → CY/ F2 → CK Rc → Rd/CMc1 → F2/CMc → CK	PDB	28	5.0	144	2.47 (g L^−1^)	[69]
Enzyme recombinant expressed in *E. coli*							
*bgpA* coding for β-glucosidase from *Terrabacter ginsenosidimutans*	Rb1 → Gyp XVII → Gyp LXXV → CK	SD	45	7	N/A	N/A	[62]
*bgp3* coding for β-glucosidase from *Microbacterium esteraromaticum*	Rb1→ Rd → CK	SD	40	7	1	77% (0.46 mg/mL)	[63]
	Rb2 → CY → CK	SD	40	7	12	0.1 (mg mL^−1^)	[64]
β-glucosidase from *Bifidobacterium breve* ATCC 15700	Rd → F2 → CK	CB/PB	35	5.0	12	96%	[65]
β-glucosidase from *Sulfolobus solfataricus*	Rb1 or Rb2 → Rd → F2 → CK, Rc → CMc → CK	Z buffer	85	5.5	12	1.63 (mg mL^−1^)	[70]
	Rb1→ Rd→ CK, Rb2→ CY→ CK	CB	85	5.5	3	0.53 (mg mL^−1^)	[66]
β-glucosidase from *Pyrococcus furiosus*	Rb1, Rb2, or Rc → Rd → CK	CB	95	5.5	1	2.010 (mg L^−1^ h^−1^)	[67]
Cytolase PCL5	Rb3 → Rd → F2 → CK	N/A	55.36	4.3	78.05	2.068 (mg mL^−1^)	[71]
Enzyme recombinant expressed in *Lactococcus lactis* NZ9000							
β-glucosidase genes (*BglPm* and *BglBX10*) from *Paenibacillus mucilaginosus* and *Flavobacterium johnsoniae*	Rb1 → Rd → F2 → CK	SD	N/A	7.0	36	70%	[72]
Enzyme recombinant expressed in *Pichia pastoris*							
β-glucosidase from *Sulfolobus solfataricus*	Rb1 → Rd → F2 → CK	AB	80	6.0	30	82.5%	[73]
Intestinal bacterial hydrolysis							
*Eubacterium* sp. A-44	Rb1 → Rd → F2 → CK	GAM	37		24	9.6 nmol min^−1^ mg^−1^	[74]
					7	4.8 ng mL^−1^	
					15	83.4 ng mL^−1^	
Human gut bacteria	Rb1 → Rd → F2 → CK	BHI	37		36	186.9 (µg mL^−1^)	[75]
*Bifidobacterium* K-103	Rc → Rd → F2 → CK	GAM or TSTA	37		24	62.3 (µg mL^−1^)	[76]
*Bifidobacterium* K-506	Rc → Mb → F2/Mc → CK	GAM or TSTA	37		24	6.5 (µg mL^−1^)	
*Bacteroides* JY-6	Rc → Mb → F2/Mc → CK	GAM or TSTA	37		24	6.7 (µg mL^−1^)	
CK production from endophytes							
*Panax ginseng*							
*Arthrinium* sp. GE 17-18	Rb1 → Rd → F2 → CK	PDA	30		N/A	N/A	[77]
*Panax notoginseng*							
*Fusarium oxysporum* YMF1.02670	Rb1 → Rd → F2 → CK	PDA	28		12 days	4 mg	[78]
*Platycodon grandiflorum*							
*Luteibacter* sp. JG09	Rb1 → Rd → F2 → CK	LB	30		7 days	66.34%	[79]

Note: MDES, medium-deep eutectic solvent; SA, sodium acetate; SD, sodium phosphate; FB, formate buffer; AB, acetate buffer; CB, citrate buffer; PB, phosphate buffer; PDA, potato dextrose agar; PDB, potato dextrose broth; LB, liquid broth; GAM, general anaerobic medium; BHI, brain heart infusion; TSTA; tryptic soy broth containing 0.01% sodium thioglycolate and 0.1% ascorbic acid; and N/A, not available.

The lack of post-translational modification and low intracellular expression in *E. coli* led to a limit on the production of CK. Consequently, there is a need to explore alternative microbial hosts for ginsenoside production. Recently, *Pichia pastoris* (*P. pastoris*) and *Lactococcus lactis* have been used as microbial hosts for β-glucosidase expression [72,73]. Both strains are generally regarded as safe (GRAS) microbes. While lactic acid bacteria (LAB) possess probiotic characteristics, *P. pastoris* exhibits the ability for post-translational modification and rapid growth at high cell densities. Therefore, these strains are a suitable expression system for heterologous protein production. In the case of lactic acid bacteria, *L. lactis* NZ9000, carrying β-glucosidase genes (*BglPm* and *BglBX10*) from *Paenibacillus mucilaginosus* and *Flavobacterium johnsoniae*, demonstrated the conversion of up to 70% of Rb1 to CK [73]. In another case, *P. pastoris* was used as a microbial host for the expression of a thermostable β-glucosidase from *Sulfolobus solfataricus*. The recombinant SS-bgly expressed in *P. pastoris* achieved an 82.5% conversion rate of Rb1 to CK (Table 2) [73]. These results indicated that lactic acid bacteria and *P. pastoris* could be potential candidates for the industrial production of the rare ginsenoside CK.

### 3.3. Biotransformation by the Human Intestinal Bacteria

Although ginseng plants primarily contain major ginsenosides, the human body could obtain CK via oral administration of ginsenosides. A study was conducted to investigate the concentration of ginsenosides and CK in human plasma, involving 11 healthy Korean adults who consumed red ginseng extract for 2 weeks [7]. The results showed a decrease in the concentration of Rg3 while CK and its metabolites increased over time. This slow absorption suggests that CK and its metabolites can be absorbed in the intestine, with intestinal bacteria playing a crucial role in generating CK from major ginsenosides. The transformation process of ginsenosides by the gut microbiota is extremely complex, with 15 different metabolites from protopanaxadiol saponins.

Although major ginsenosides such as Rb1, Rb2, and Rc, possess high solubility, they have low membrane permeability and are susceptible to degradation [80]. Intestinal bacterial hydrolysis not only plays a significant role in metabolic function but also improves the absorption and stability of ginsenosides. The conversion pathway, Rb1→ Rd → F2 → CK, is known as one of the ubiquitous approaches to biotransformation by intestinal bacteria. Various bacterial species, including *Eubacterium* sp. A-44 isolated from rat or human gut bacteria such as *Streptococcus* sp. and *Bifidobacterium* sp., showed the ability to transform Rb1 to CK through geniposide-hydrolysing β-D-glucosidase activity [74,75]. Similarly, Hasegawa et al., [81] isolated *Prevotella oris* strains from human fecal samples, which were found to have the ability to hydrolyze ginsenoside Rb1 into CK. Studies have also identified the transformation pathway of Rd → F2 → CK through selective hydrolysis of the C-3 in Rd using β-D-glucosidase enzyme [65]. Interestingly, another pathway via Rb1 → G-XVII → G-LXXV/F2 → CK was investigated when pooled gut bacteria were incubated anaerobically with Rb1. It was observed that the rate of the pathway was rapid and the percentage of conversion from G-XVII to G-LXXV was minor in comparison with the conversion of G-XVII to F2. These findings indicated that human gut bacteria could digest glucose residue at the C-20 position in addition to Rb1, and other major ginsenosides are also utilized by microbiota for conversion into CK. The bacteroide HJ-15 transforms Rc into CK via ginsenosides Mb and Mc (Rc → Mb → Mc → CK) (Figure 3; Table 2) [76]. In summary, bacteria utilize stepwise sugar cleavage reactions to transform various ginsenosides [82].

A study on the plasma levels of compounds in 15 individuals found significant variation in the concentrations of Rd and CK among subjects. Some individuals had much higher levels of Rd and CK than others in the study. The data recorded on the first and fifteenth days showed that this difference was not dependent on the method of extract use: either a single dose or multiple days in a row. This difference suggests that some individuals have a higher capacity to convert ginsenosides to Rd and CK [83]. The absorption rate of CK in the blood is directly linked to the body’s ability to convert it [84]. In mice, this ratio is also influenced by the diet, including prebiotics [85]. Furthermore, the absorption rate of CK also varies depending on the species. The concentration in the plasma and AUC of CK in mice is 5–6 times higher than in rats, although there is no significant difference in half-life or average residence time between the two species. Analysis of bacterial composition in fecal samples revealed that groups of *Bacteroides* sp., *Eubacterium* sp., and *Bifidobacterium* sp. in mice have lower proportions and activity compared to humans [86]. It has been reported that humans with a higher proportion of *Bacteroides* sp. in their gut microbiota have six times higher metabolic activity of compound K than those with a lower proportion of *Bacteroides* sp. [71]. The study also highlighted the high diversity and richness of the group with strong conversion ability, particularly in the dominant groups *Firmicutes*, *Bacteriodetes*, and *Tenericutes* [87].

Overall, the gut microbiota is healthy. However, gut bacterial enzymes are influenced by many factors, particularly dietary habits. Enzyme activity is not dependent on gender or age but varies between individuals, impacting their ability to convert ginsenosides [88]. These reports have shown that the low absorption of ginsenoside metabolites is significantly dependent on the composition and metabolic activity of the gut microbiota.

### 3.4. Biotransformation by Endophytes

As mentioned previously, CK is a minor ginsenoside that cannot be extracted in large amounts from the natural ginseng plant. Therefore, several studies have focused on transforming major ginsenosides to CK through different methods such as hydrolysis, enzymatic biotransformation, microbial transformation, etc. Inside microbial transformation, biotransformation by endophytes is an efficient method due to its low price, high accuracy, selectivity, and environmental protection [89]. Endophytes are microorganisms inside vascular tissues and intercellular spaces in plant tissues that have the function of infiltrating healthy plant tissues without causing any disease for the plants [90]. Based on their unique living conditions and extended coexistence with their hosts, endophytes have developed distinct adaptations to maintain a consistent symbiosis. They can also synthesize a variety of extracellular enzymes for the manufacture of secondary metabolites. Thus, to produce more active compounds, complicated processes involving endophytes have been applied to the biotransformation of region- and stereo-selective synthesis to converse natural compounds [91].

In recent years, numerous studies have focused on exploring different endophytes that could participate in the biotransformation of major ginsenoside to minor ginsenoside, including CK. It has been reported that the glucosidase of endophytes *Fusarium* sp. YMF1.02670 or YMF1.02193 could deglycosylate the major ginsenoside Rb1 to CK [78]. Among 32 β-glucosidase-producing endophytes, extracted endophyte bacteria JG09 defined as *Luteibacter* sp. from *P. grandiflorum* is capable of efficiently converting CK from the major ginsenosides Rb1, Rb2, and Rc. To enhance the yield of CK, the optimal conditions of the fertile process and the content of saponins were evaluated. Endophyte JG09 was found to hydrolyze Rb1, Rb2, and Rc to CK by distinct β-glucosidase in the following pathways: Rb1 → Rd → F2 → CK; Rb2 → CO → CY → CK; Rc → CMc1 → CMc → CK; and Rd → F2 → CK. After 7 days, the highest yield of CK was recorded at 66.34% (Figure 3; Table 2) [79]. Similarly, the extracted β-glucosidase-producing endophyte from P. ginseng also participated in the biotransformation of Rb1 to CK through the hydrolyzation method in the following sequence: Rb1 → Rd → F2 → CK [77]. Moreover, a different study also represented a high percentage of transformation from Rb1 to CK, with the contribution of endophytes *Fusarium oxysporum* and *Coniochaeta* sp. extracted from ginseng and *P. notoginseng* [92]. In another study, using β-glucosidase isolated from *Armillaria mellea* mycelium, Rb2 was hydrolyzed and transformed into CK via the catalytic pathway Rb2 → CO → CY → CK [93]. These studies have showcased the significant control that endophytes exert in the biotransformation of major ginsenosides to CK. The advancement of fermentation, extraction, purification, characterization, and bioassay techniques has also contributed to the improvement of the biotransformation process [94]. Moreover, the fermentation process is speedy, efficient, and commercially sustainable, with plenty of room for manipulation through the addition of precursors, elicitors, specialized enzymes, and modifiers for the effectively increased synthesis of bioactive chemicals. However, there are ongoing challenges in maintaining the biotransformation by endophytes that should be considered.

## 4. Metabolically Engineered Microbes

**Figure 4:**
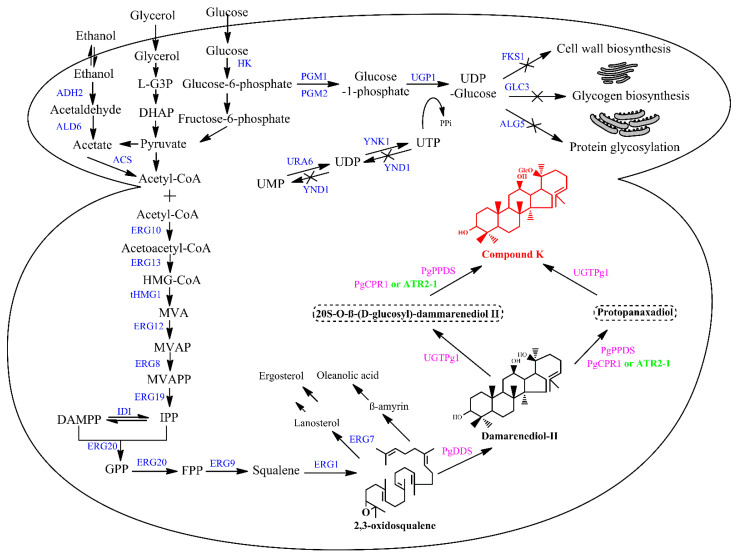
The proposed biosynthetic pathway for CK production in engineered yeasts. Blue: yeast native enzymes; purple: *P. ginseng* enzymes, and green: *A. thaliana* enzyme. Intermediates: HMG-CoA, β-Hydroxy β-methylglutaryl-CoA; DMAPP, dimethylallyl pyrophosphate; IPP, isopentenyl pyrophosphate; FPP, farnesyl diphosphate; MVA, mevalonate; MVAP, mevalonate 5-phosphate; MVAPP, mevalonate 5-pyrophosphate; UTP, uridine triphosphate; UDP, uridine diphosphate; and UMP, uridine monophosphate. Enzymes: ACS: acetyl-CoA synthase; ADH2, alcohol dehydrogenase; HK, hexokinase; ALD6, acetaldehyde dehydrogenase; ERG10, acetyl-CoA C-acetyltransferase; ERG13, HMG-CoA synthase; HMG, 3-hydroxy-3-methylglutaryl-CoA reductase; ERG12, mevalonate kinase; ERG8, phosphomevalonate kinase; ERG19, diphosphomevalonate; IDI, isopentenyl diphosphate-isomerase; ERG20, farnesyl diphosphate synthase; ERG9, squalene synthase; ERG1, squalene epoxidase; ERG7, lansterol synthase; CPR, cytochrome P450 reductase; PPDS, protopanaxadiol synthase; UGT, UDP-glycosyltransferase; PGM: phosphoglucomutase 2; FKS1, 1,3-β-D-glucan synthase; GLC3, glycogen-branching enzyme; and ALG5, glycosyltransferase on N-linked glycosylation.

Engineering microorganisms is a sustainable and promising approach for the production of plant-derived secondary metabolites to accelerate industrialization. Synthetic biology and metabolic engineering have made significant progress in producing high-value compounds. For example, flavonoids and stilbenes are produced by *E. coli* [95], taxol is produced by *E. coli* and *Saccharomyces cerevisiae* [96,97], and artemisinin is produced by *S. cerevisiae* [98]. To date, the highest terpenoid titers have been achieved using *E. coli* and *S. cerevisiae*. Both model microbes are known as GRAS organisms with well-characterized microbial cell factories. They are not only easy to cultivate and grow fast but also produce high efficiency and productivity of terpenoids [99]. The toxic accumulation of CK negatively affects the growth of *E. coli*, and expressing plant cytochrome P450s in *E. coli* is challenging [60]. By contrast, yeasts, including *S. cerevisiae* and *Yarrowia lipolytica*, possess redox systems that allow tailoring enzymes such as CYP450s and glycosyltransferase to further modify the core structure of terpenoids. As a result, yeast cells can produce CK as well as other terpenoids [18,100]. According to the advantages and characteristics of yeasts, metabolic engineering strategies provide various approaches to producing CK. First, the common approach is the overexpression of heterologous genes in *S. cerevisiase*. The genes of CK biosynthesis from squalene involve endogenous squalene epoxidases 1 (*ERG1*), dammarenediol synthase (*PgDDS*) and UDP-glycosyltransferase (*UGT71A28*) from *P. ginseng*, and a cytochrome P450 (*CYP716A47*) from *P. ginseng* co-expressed with an NADPH-cytochrome P450 reductase (*ATR2-1*) from *Arabidopsis thaliana*. In this pathway, ERG1 catalyzes the conversion of squalene to 2,3-(S)-oxidosqualene. Then, 2,3-(S)-oxidosqualene, an intermediate from the mevalonate pathway in *S. cerevisiae*, is converted to dammarenediol II (DD II) by catalyzing PgDDS. Next, UGT71A28 catalyzes the formation of DMG from DD II under the availability of UDP-Glucose in the cells. After that, CYP716A47 fused-ATR2-1 is catalyzed as a monooxygenase to the formation of CK from DMG. On the other hand, DD II is converted to PPD by CYP716A47 fused-ATR2-1 and forms CK from PPD by catalyzing UGT71A28 (Figure 4). Noticeably, UGT71A28 acts as a stereospecific and regioselective glycosyltransferase, which transfers glucose residues to the C-*20S*-OH of PPD. As a result, engineered *S. cerevisiae* BK1 harboring biosynthesis pathway genes produced 155.4 μg L^−1^ of CK using glucose as a carbon source (Table 3) [101].

Secondly, the control of gene expression has a significant effect on the optimization of cell factories. Promoter sequences encode the level of gene expression by regulating the transcription in yeast *S. cerevisiae*. Making a rational design of the promoter is one of the most effective approaches for controlling gene expression. Constitutive and inducible promoters have been widely used for gene expression in recent years. Constitutive promoters are constantly active under various cultural conditions. Well-known examples in yeasts include constitutive promoters of ribosomes (*P_RP_*, cytoplasmic ribosomal protein; *P_ribi_*, ribosome biogenesis; and *P_snoRNA_*, small nucleolar RNA genes) [107], promoters for genes encoding the cellular translational machinery (*P_TEF1_*, and *P_TEF2_*, translation elongation factor EF-1α and EF-2 α, respectively) [108], and constitutive promoters of glycolytic genes (*P_GAP_*, glyceraldedyde-3-phosphate dehydrogenase; *P_GPM1_*, phosphoglycerate mutase; and *P_ADH1_*, alcohol dehydrogenase) [109]. For example, the expression of genes related to the MVA pathway under the control of a constitutive promoter in *S. cerevisiae* ZW-F1-17, including *ERG20* under the control of *P_GPM1_*, *EGR9*, *tHMG1*, and *UGTPg1* under the control of *P_GK1_*, *PgERG1*, and *CYP716A53v2* under the control of *P_TEF1_* and *PgCPR1* under the control of *P_TDH3_*, resulted in the production of 7.5 mg L^−1^ of CK [102]. Due to the strong characteristics of these promoters, the expression of certain enzymes in metabolic pathways leads to the production of toxic by-products, thus reducing cell growth [110]. Furthermore, the flux regulation of the central carbon metabolism and the demand for energy (ATP) and redox cofactors (NADPH) lead to an increase in metabolic burden for yeast cells [111]. Therefore, inducible promoters are a sustainable approach to the replacement of strong constitutive promoters under dynamic cultural conditions. Carbon source-dependent promoters allow activation of a biosynthesis pathway after the host growth phase has been completed [108]. For example, in the replacement of constitutive promoters *P_GPM1_* and *P_TEF1_* by the galactose inducible promoters *P_GAL1_* and *P_GAL10_* in engineered *S. cerevisiae* AK1, CK was produced 1.57-fold higher than a BK1 strain from galactose with a yield of 244.8 μg L^−1^ [101].

Thirdly, the identification and overexpression of key rate-controlling enzymes in the biosynthesis pathway are promising approaches to improving the target products. In the MVA pathway, 3-hydroxy-3-methylglutaryl-coenzyme A (HMG) reductase (HMG1, HMG2) catalyzes the conversion of HMG to MVA. This enzyme is identified as the rate-limiting enzyme because it shows the ability to inhibit post-transcriptional feedback in the MVA pathway [112]. In order to address this bottleneck, the removal of the N-terminal transmembrane sequence, which encodes membrane-binding activity, has commonly been used to enhance enzyme activity. In addition, overexpression of *tHMH1* under control of *P_ADH1_* along with a semi-dominant mutant allele of a global transcription factor for sterol biosynthesis (UPC2.1) under control of *P_ADH1_* has significantly improved CK production. The engineered *S. cerevisiae* AKE and BKE produced a 5-fold increase in the yield of CK, with 1424.8 and 802.2 μg L^−1^, respectively [101]. Similarly, the inefficiency of UDP-sugar, the donor sugar moieties of glycosyltransferase enzymes, is one of the major limiting factors for the production of CK in yeast cells. An engineered strain for CK production was developed by overexpressing a UDP-glucose biosynthetic pathway in PPD-producing *S. cerevisiase* WLT-MVA5. The UDP-glucose biosynthetic pathway included UDP-glucose glucosyltransferase (*UGP1*) from *P. ginseng*, phosphoglucomutase 2 (*PGM2*), and UTP-glucose-1-phosphate uridylyltransferase 1 (*UGP1*) from *S. cerevisiae* W303-1a. In this pathway, glucose-6-phosphate is converted to glucose-1-phosphate by PMG2. Then, UGP1 catalyzes the conversion of glucose-1-phosphate to UDPG. Finally, the formation of CK from PPD is carried out through the catalyzing of UGT1. After successful overexpression of three genes, a 79.81% increase in CK production and a 183.20% enhancement in the rate of conversion were achieved (Table 3) [103].

Fourthly, in addition to overexpression of the genes involved in UDP-glucose biosynthesis, reducing UDP-glucose consumption is an important approach to preserving and improving the available UDP-glucose in *S. cerevisiae.* Overexpression of *URA6* along with *YNK1* or the deletion of *YND1* led to improved UTP, a metabolic intermediate of UDP-glucose. While URA6 is catalyzed to produce UDP from UMP, YNK1 is converted from UMP to UTP. On the other side, UTP could be dephosphorylated by YND1 to synthesize UDP and UMP. At this stage, the combined overexpression of the genes *PGM2*, *UPG1*, *URA6*, and *YNK1* in engineered *S. cerevisiae* WPK8 or the combined overexpression of the genes *PGM2* and *UPG1* along with the deletion of *YND1* in engineered *S. cerevisiae* WPK8 resulted in an improvement in the CK titer (Figure 4) [104]. In theory, UDP-glucose could not only be converted to intermediate molecules through enzymatic reversible reactions but also be present in many yeasts’ metabolic processes, including glycogen biosynthesis, protein glycosylation, and cell well biosynthesis [113,114]. Therefore, knockdown and knockout of the genes encoding the consumption pathway of UDP-glucose are required for improving CK production. However, it has been demonstrated that the deletion of *FKS1* (encoding for yeast 1,3-β-D-glucan synthase, which catalyzes glucan chain elongation using a glucose donor from UDP-glucose on the cell wall biosynthesis) and *GLC3* (encoding for glycogen-branching enzyme) did not improve CK production in engineered *S. cerevisiae* WPK10 and WPK11, respectively (Figure 4) [115,116]. Conversely, the deletion of *ALG5* (encoding for a glycosyltransferase on N-linked glycosylation of protein in yeast) resulted in an increase of 12% of CK tilter compared to the non-mutant strain, which is the highest reported yield to date with 5.74 g L^−1^ (Table 3) [104]. It is possible that UDP-sugar is used as a compensatory mechanism in a network of biochemical reactions.

Fifthly, yeast cells consist of various subcellular compartments, such as the endoplasmic reticulum, mitochondria, peroxisomes, vacuole, and cytosol. Each subcellular compartment exhibits a unique physiochemical environment with different enzymes, cofactors, and metabolites [117,118]. Modifying subcellular compartments in engineered yeasts provides novel strategies to produce CK. Interestingly, this strategy could achieve up to 5 g L^−1^ of CK. In order to engineer storage organelles (lipid droplets) of PPD substrate from DD II, the yeast PLN1 protein was expressed in the normally endoplasmic reticulum (ER), which is the localization of cytochrome P450 enzymes PPD synthase (PPDS). It was demonstrated that the change in the ratio of volume and surface area of LDs led to a decrease in the conversion from DD to PPD, while the alternative morphology of LDs showed the effects on their storage capacity [105].

Noticeably, one of the greatest challenges of industrial production is the cost of raw materials. While non-renewable fossil raw materials for energy and commercial products are rapidly becoming a global crisis, the application of renewable carbon sources has been developed as a recent national and global strategy [119]. Currently, glucose is known as the main renewable carbon source for microbial production. However, there are various other types of renewable carbon sources available, such as agricultural and forestry residues, industrial by-products, or non-food biomass, which could be used as low-cost feedstock to produce commercial products. Moreover, these raw materials do not compete with food and feed chains. Therefore, alternative carbon source utilization is a sustainable approach to apply to microbes for bioproduction [120,121]. In the case of CK production, glycerol and ethanol are used as carbon sources in engineered *S. cerevisiase*. Glycerol, being a byproduct of the biodiesel industry, is a low-cost and abundant product. Moreover, there are several other advantages to using glycerol as a carbon source in microbial production. Firstly, glycerol provides more reducing equivalents compared to sugars. The amount of reducing equivalents from glycerol to phosphoenolpyruvate or pyruvate is 2-fold higher than the conversion from glucose [122]. Secondly, since glycerol does not show activity on the permeable membrane, it can increase the stabilizing enzyme conformation on the cell membrane. Furthermore, glycerol acts as a chaperone for protein folding [123,124]. It has been reported that glycerol increases UDPG pyrophosphatase activity and the accumulation of UDPG when *S. cerevisiae* is cultured in glycerol [125]. As a result, the production of CK from engineered *S. cerevisiae* WLT-MVA5 cultured in YPD medium containing 20% glycerol was 45.68% higher than that from this strain cultured in YPD medium containing 20% glucose [103]. Noticeably, *S. cerevisiase* produces ethanol during fermentation, and then ethanol is used as a carbon source when glucose becomes depleted [126]. Since ethanol facilitates the formation of PPD, a mixture of glycerol and ethanol was used, enabling CK production of 1.7 g L^−1^ in the 5-L bioreactor fed fermentation (Table 3) [103].

Recently, the non-conventional oleaginous yeast *Y. lipolytica* has been considered a promising host for the production of lipid-based oleochemicals [127]. Like *S. cerevisiase*, *Y. lipolytica* is known as a eukaryote cell with “GRAS” status. Moreover, the genome of *Y. lipolytica* has been well sequenced and is suited for genetic manipulation [128]. However, there are many advantages of *Y. lipolytica* in the production of hydrophobic compounds compared to *S. cerevisiase*. Firstly, unlike the Crabtree-positive yeast *S. cerevisiae*, *Y. lipolytica* is Crabtree-negative and possesses a respiratory metabolism with a robust energy supply system, enabling higher biomass yields in fermentation processes [106]. Therefore, *Y. lipolytica* metabolism avoids carbon loss through excretion of acetate and ethanol. Moreover, unlike *S. cerevisiae*, *Y. lipolytica* can grow with high growth rates on various renewable carbon sources, such as glycerol, pentose, waste oil, fatty acids, and C1 carbon sources. This characteristic may provide a promising industrial host for the economic production of high-value compounds [129]. Importantly, the metabolic traits of *Y. lipolytica* include high acetyl-CoA flux, which is known as a key precursor of the MVA pathway [130]. These traits are thus of interest for the application of metabolic engineering and synthetic biology to the synthesis of terpenoids, such as monoterpenoids (limonene and linalool) [131,132], sesquiterpenoids (α-farnesene and (+)-nootkatone) [37,133], and tetraterpenoids (β-carotene and lycopene) [134,135]. However, engineering *Y. lipolytica* for the production of triterpenoids has been rarely performed; especially since *Y. lipolytica* cannot be directly synthesized to CK due to its lack of three enzymes, including DDS, CYP450s, and UGT. To increase the metabolic flux of 2,3-Oxidosqualene and promote the accumulation of the final product, overexpression of key genes in the MVA pathway and heterologous expression of lacking genes have been investigated.

## 5. Conclusions and Future Perspectives

CK exhibits various important biological and pharmaceutical properties, including antitumor, anti-cancer, anti-diabetic, anti-skin aging, hepatoprotective, and neuroprotective effects. The research of CK not only focuses on expanding various biological activities but also aims to understand its multiple molecular mechanisms. Although ginseng of the *Araliaceae* family commonly produces protopanaxadiol and protopanaxatriol ginsenoside, CK is still absent from natural ginseng. The various processes used for CK biosynthesis include chemical synthesis, enzymatic reactions, microbial transformation, and metabolic engineering. Chemical synthesis is not only rare but also has low yields. Chemical methods use toxic agents and organic solvents, which are hazardous to human health and the environment. While green chemistry has reduced and eliminated the use of hazardous substances, biological approaches have shown many advantages for the production of CK.

Microbial hosts, including human intestinal bacteria, endophytes, and industrial microbes, have been shown to be the most important cell factories for CK production. While endophytes are known as a promising source of ginsenosides, biotransformation has also been carried out by intestinal bacteria. The discovery of novel endophytes from *ginseng* plants would provide potential approaches to producing CK. Moreover, the investigation of β-glucosidase-produced endophytes is necessary to increase the amount of biotransformation for the production of CK. Similarly, further investigation is required to understand the distribution, biodiversity, and composition of gut microbiota. The β-glucosidase from endophytes and intestinal bacteria could be supported by the genes in engineered strains, such as *E. coli* or/and *S. cerevisiae*. This approach provides various sources of enzymes for in vitro reactions, which may improve the efficient production of CK.

Recently, the whole genome of ginseng plants and endophytes has been investigated through omics tools. Omics tools, including next-generation sequencing, metagenomics, transcriptomics, proteomics, and metabolomics, have been applied to identify genes encoding for three enzymes crucial for CK biosynthesis: OSCs, CYP450s, and GTs. However, the functional genes and enzymes from omics data are still limited. The reason is that the characterized OSCs, CYP450s, and GTs are most likely sourced from *P. ginseng*. The identified genes encoding for three enzymes from other ginseng plants have not been well understood yet. Furthermore, omics data from the ginseng endophytes is still lacking. Therefore, the development of omics data from the ginseng plants and their endophytes is required for further studies.

Noticeably, metabolic engineering assisted-synthetic biology provides a promising approach to producing CK in endophytes and engineered microbes. Various metabolic engineering strategies on yeast strains, such as heterologous gene expression, enzyme engineering, codon optimization, copy number multiplication, subcellular localization, balancing, and increasing metabolic flux, have been applied to achieve the efficient production of CK. Importantly, the development of synthetic biology tools, including RNA interference (RNAi) and CRISPR-Cas systems, have emerged as a powerful tool for genome editing, which allows knock-down, knock-out, knock-in, and fine-tuning of genes from the CK biosynthetic. While synthetic biology tools have been highly applied for genomic editing on *S. cerevisiae*, *Y. lipolytica* and *P. pastoris* have not been much used as hosts for CK production. Therefore, these two non-conventional yeasts could be engineered by synthetic biology tools to improve the productivity of CK.

## Figures and Tables

**Table 1:** Classification of four types of ginsenosides.

Structure	Name	R1	R2	R3	R4
Protopanaxadiol (PPD) Type					
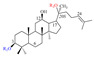	CK	H	-	Glc	-
	CO	Glc	-	Glc^6^-Ara(p)	-
	CY	H	-	Glc^6^-Ara(p)	-
	CMc	H	-	Glc^6^-Ara(f)	-
	CMc1	Glc	-	Glc^6^-Ara(f)	-
	CMx	H	-	Glc^6^-Xyl	-
	CMx1	Glc	-	Glc^6^-Xyl	-
	Gyp XVII	Glc	-	Glc^6^-Glc	-
	Gyp LXXV	H	-	Glc^6^-Glc	-
	F2	Glc	-	Glc	-
	Rh2	Glc	-	H	-
	Rg3	Glc^2^-Glc	-	H	-
	Rd	Glc^2^-Glc	-	Glc	-
	Rc	Glc^2^-Glc	-	Glc^6^-Ara(f)	-
	Rb3	Glc^2^-Glc	-	Glc^6^-Xyl	-
	Rb2	Glc^2^-Glc	-	Glc^6^-Ara(p)	-
	Rb1	Glc^2^-Glc	-	Glc^6^-Glc	-
	Ra1	Glc^2^-Glc	-	Glc^6^-Ara(p)^4^-Xyl	-
Protopanaxatriol (PPT)-type					
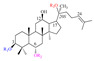	F1	OH	H	Glc	-
	Rh1	OH	Glc	H	-
	Rg2	OH	Glc^2^-Rha	H	-
	Rg1	OH	Glc	Glc	-
	Rf	OH	Glc^2^-Glc	H	-
	Re	OH	Glc^2^-Rha	Glc	-
	PPT	OH	H	H	-
Ocotillol-type					
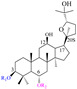	Vinaginsenoside R1	OH	Ac-Glc^2^-Rha	-	-
	Majonoside R2	OH	Glc^2^-Xyl	-	-
Oleanolic acid type					
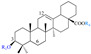	R_OA_	GlcUA-Glc	-	-	Glc^6^-Glc
	R_O_	GlcUA-Glc	-	-	Glc

Note: C: compound; Ac: acetyl; Ara(f): α-L-arabinofuranosyl; Ara(p): α-L-glucopyranosyl; Glc: glucose; Gyp: gypenoside; xyl: β-D-xylopyranosyl; GlcUA: glucuronic acid; Rha: α-L-rhamnopyranosyl.

**Table 3:** List of the engineered yeasts for CK production under shake-flask/fed-batch conditions with appropriate medium at 30 °C.

Strains	Related Gene Cassettes in Biosynthesis Pathway	Titer	Major Media	Carbon Source	Cultivation Condition	Ref.
*Saccharomyces cerevisiae*						
BK1 (BA21)	*ERG1*, *PgDDS*, *PgCYP716A47*, *AtATR2-1*, *UGT71A28*	155.4 (μg L^−1^)	SC	Glucose	Shake-flask	[101]
BKE	*AtATR2-1*, *UGT71A28*, *PgCYP716A47*, *ERG1*, *PgDDS*, *tHMGR-UPC2.1*	802.1 (μg L^−1^)				
AKE	*ERG1*, *PgDDS*, *PgCYP716A47*, *AtATR2-1*, *UGT71A28*	243.8 (μg L^−1^)		Galactose		
AK1	*AtATR2-1*, *UGT71A28*, *PgCYP716A47*, *ERG1*, *PgDDS*, *tHMGR-UPC2.1*	1424.8 (μg L^−1^)				
ZW-F1-17	*ERG20*, *PgERG1*, *ERG9*, *tHMG1*, *CYP716A53v2*, *PgCPR1*, *UGTPg1*	7.5 (μg L^−1^)	SC	Glucose	Shake-flask	[102]
WLN-3	*DS*, *PPDS-ATR1*, *ERG1*, *tHMGR*, *ERG9*, *ERG20*, *ERG10*, *ERG13*, *ERG12*, *ERG8*, *ERG19*, *IDI1*, *NCP1*, *ACSseL641P*, *TetR*, *PGM2 and UGP1*, *PgUGP1*	263.94 (mg L^−1^)	YPD	Glucose	Shake-flask	[103]
WLN-3	*DS*, *PPDS-ATR1*, *ERG1*, *tHMGR*, *ERG9*, *ERG20*, *ERG10*, *ERG13*, *ERG12*, *ERG8*, *ERG19*, *IDI1*, *NCP1*, *ACSse_L641P_*, *TetR*, *PGM2 and UGP1*, *PgUGP1*	384.52 (mg L^−1^)	YPDG (20%)	Glucose, glycerol	Shake-flask	
WPK12	*ERG10*, *ERG13*, *tHMG1*, *ERG12*, *ERG8*, *ERG19*, *IDI1*, *ERG20*, *ERG9*, *ERG1*, *PgDDS*, *PgPPDS*, *PgCPR1*, *synUGTPg1*, *PGM2*, *URA6*, *YNK1*, *ΔAGL5*	5.74 (g L^−1^)	YPD	Glucose	Fed batch	[104]
LPTA-M	*ERG12*, *tHMG1*, *ERG13*, *ERG10*, *ERG8*, *ERG19 IDI1*, *AtSQS2*, *ERG1*, *SmFPS*, *SynPgPPDS*, *ATR1*, *PLN1*	5.0 (g L^−1^)	SD	Glucose	Shake-flask	[105]
*Yarrowia lipolytica*						
YL-MVA-CK	*tHMG1*, *ERG9*, *ERG20*, *opDS*, *PPDS* linker2-*ATR1*, *UGT1*	161.8 (mg L^−1^)	YPD	Glucose	Fed batch	[106]

## Data Availability

This study did not report any data that needs to be available.
